# Structural Origins of Viscosity in Imidazolium and Pyrrolidinium Ionic Liquids Coupled with the NTf_2_^–^ Anion

**DOI:** 10.1021/acs.jpcb.3c02604

**Published:** 2023-07-11

**Authors:** Raphael Ogbodo, Waruni V. Karunaratne, Gobin Raj Acharya, Matthew S. Emerson, Mehreen Mughal, Ho Martin Yuen, Nicole Zmich, Shameir Nembhard, Furong Wang, Hideaki Shirota, Sharon I. Lall-Ramnarine, Edward W. Castner, James F. Wishart, Andrew J. Nieuwkoop, Claudio J. Margulis

**Affiliations:** † Department of Chemistry, The University of Iowa, Iowa City, Iowa 52242, United States; ‡ Department of Chemistry and Chemical Biology, Rutgers University, Piscataway, New Jersey 08854, United States; § Department of Chemistry, Queensborough Community College-CUNY, Bayside, New York 11364, United States; ∥ Chemistry Division, Brookhaven National Laboratory, Upton, New York 11973-5000, United States; ⊥ Department of Chemistry, Chiba University, Chiba 263-8522, Japan

## Abstract

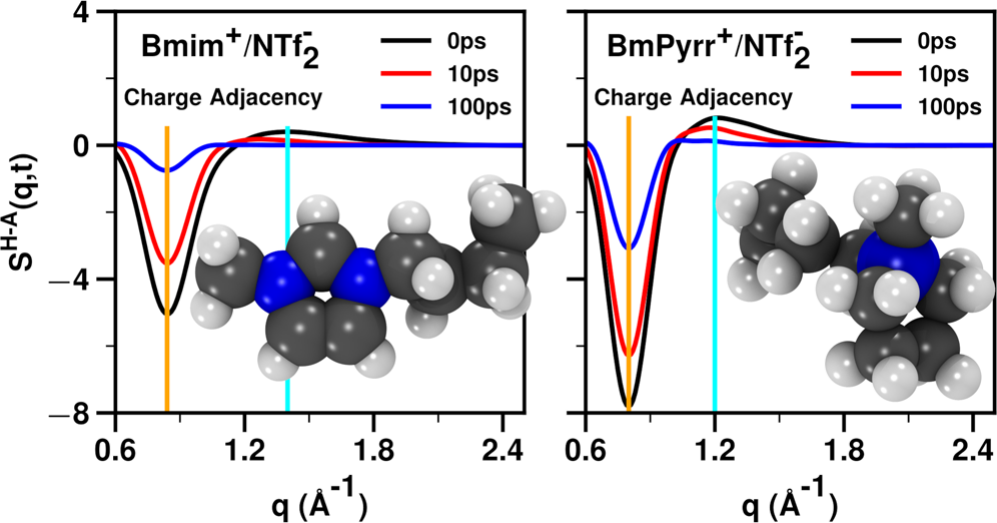

Ionic liquid viscosity is one of the most important properties to consider for practical applications. Yet, the connection between local structure and viscosity remains an open question. This article explores the structural origin of differences in the viscosity and viscoelastic relaxation across several ionic liquids, including cations with alkyl, ether, and thioether tails, of the imidazolium and pyrrolidinium families coupled with the NTf_2_^–^ anion. In all cases, for the systems studied here, we find that pyrrolidinium-based ions are “harder” than their imidazolium-based counterparts. We make a connection between the chemical concept of hardness vs softness and specific structural and structural dynamic quantities that can be derived from scattering experiments and simulations.

## Introduction

**Figure 1:**
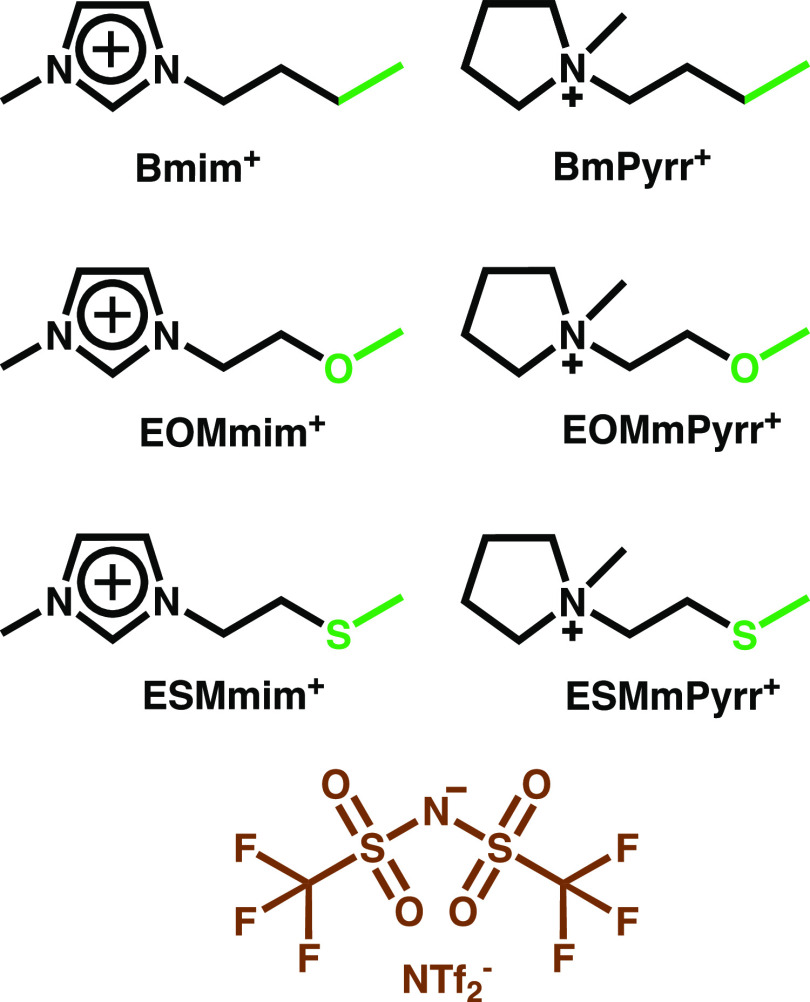
Ionic liquids comprising the imidazolium and pyrrolidinium cations paired with the NTf_2_^–^ anion. The color scheme highlights the definition used in subsequent sections for cationic-head, cationic-tail, and anion; the figure also defines our naming convention for the different species.

In contrast to conventional solvents or their mixtures, where friction in different liquid locations is expected to be homogeneous, ionic liquids show remarkable frictional heterogeneity.^1−8^ For example, small neutral probes will undergo random walks where the “step distance” very much depends on their proximity to charges. On a short time scale, even intramolecular components of ILs will show differences in mobility depending on whether these are charge-depleted or part of charge networks. Understanding the interplay between liquid structure, friction, and ultimately the macroscopic viscosity of ILs is important because of technical and economic implications. Such considerations affect applications in separations, batteries, heat transfer, and tribology, to mention just a few.^9−16^ Efforts have been underway to better understand the role that specific chemical substitutions including the introduction of ether and thioether moieties have on transport properties.^17−21^ Our experimental and computational work in this article focuses on ionic liquids (ILs) based on the popular imidazolium and pyrrolidinium cations coupled with the NTf_2_^–^ anion, which are expected to have good mobilities^22^ and reasonably low viscosities.^23−29^ We include the prototypical alkyl-tail versions of these, and also ether- and thioether-substituted analogues as shown in Figure 1. Data on density, conductivity, viscosity, and *T*_g_ are compared for all of these systems, and their structure is characterized via X-ray scattering. Simulations help us go beyond what we can measure by connecting the static structure of these ILs as well as their structural dynamics^1−4,30−37^ with the viscosity and viscoelastic relaxation. We will show that this exercise will help us further our chemical intuition on the concept of harder and softer ions. Our goal is to connect a set of physical functions and quantities, including the intensity of subcomponents of *S*(*q*), the time relaxation of subcomponents of *S*(*q*, *t*), the time-dependent Green–Kubo expressions for the viscoelastic relaxation, and the macroscopic viscosity, with simple chemical concepts. Ultimately, in the simplest possible terms, we want to answer how ion hardness manifests in structural dynamic and transport-related properties that we can measure or compute, with the idea of being able to recognize such patterns for other systems in the future.

**Figure 2:**
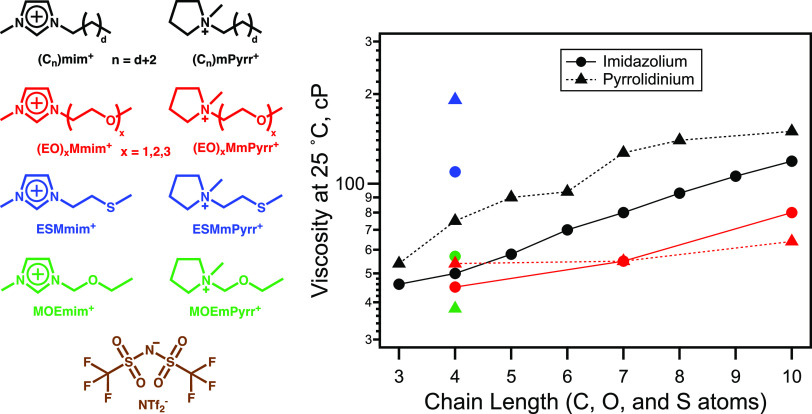
Ionic structures (left) corresponding to ILs for which the viscosities at 25 °C (calculated from VTF fits) vs chain length are plotted on the right. Colors on the left match those of the symbols on the right. Alkylimidazolium NTf_2_^–^ viscosities are from Tariq et al.^24^ Alkylpyrrolidinium NTf_2_^–^ viscosities for *n* = 3, 6, and 10 are from Jin et al.,^25^ the one for *n* = 4 is from Funston et al.,^26^ and those for *n* = 5, 7, and 8 are from Lall-Ramnarine et al.^27^ Ether imidazolium and pyrrolidinium NTf_2_^–^ viscosities with an ethylene bridge for *n* = 7 and 10, as well as for *n* = 4 for imidazolium, are from Lall-Ramnarine et al.^27^ while *n* = 4 for pyrrolidinium is from Funston et al.^28^ Ether imidazolium and pyrrolidinium NTf_2_^–^ viscosities with a methylene bridge are from Chen et al.^29^

To motivate our discussion, we note from Figure 2 that at room temperature, each member in the family of the alkylated pyrrolidinium-based ILs (C*_n_*mpyrr^+^/NTf_2_^–^) is more viscous than the corresponding imidazolium-based (C*_n_*mim^+^/NTf_2_^–^) counterpart. With limited experimental data, this also appears to be true for shorter-chain ethyl ether- and thioether-substituted cations (red and blue points, respectively). Notice that in the case of repeating ethyl ether units, or in the case where only one methylene group separates the ring and the oxygen substituent, the cation order is reversed.

As is well established,^5,27,38−46^ often ionic liquids can be described as systems with a fully percolating charge network with tails that act as spacers between charge alternating strands. In a set of prior articles,^1,2^ we explored hypotheses as to which structural motifs common to all ILs contributed the most to the viscoelastic relaxation. For example, for C_8_mim^+^/NTf_2_^–^ and C_2_mim^+^/NTf_2_^–^ kept at the same viscosity value, about 65% of the viscoelastic relaxation could be ascribed to positive-negative charge network dynamics.^1^ Even when intermediate range order (across-charge strand correlations) was significant, such as in the case of C_8_mim^+^/NTf_2_^–^, the dynamics of ions at these intermediate ranges associated with the pre-peak only modestly contributed to this relaxation by about 6%. Instead, correlations at shorter distance, what we call adjacency correlations, made up the second largest contribution to the viscoelastic relaxation. Given the importance that the charge network appears to have in defining viscosity and the viscoelastic relaxation,^1−3,34,37^ this article is set to explore differences in the static structure and its relaxation of imidazolium-based and pyrrolidinium-based ILs coupled with NTf_2_^–^; the goal is to establish simple principles that explain the observed trends across these.

## Methods

### Materials

All chemicals used in the syntheses were of reagent grade obtained from commercial sources and used as received. 1-Methylimidazole, 1-methylpyrrolidine, and 2-chloroethyl methyl sulfide were purchased from Sigma-Aldrich. Lithium bis(trifluoromethylsulfonyl)imide was obtained from IoLiTec, Inc. Dimethyl sulfoxide-*d*_6_ was purchased from Cambridge Isotope Laboratories. ^1^H and ^13^C NMR spectra were recorded on a Bruker 400 MHz NMR spectrometer with Topspin software.

### Synthesis of Thioether Chloride Salt Precursors

#### Synthesis of 1-Methyl-1-(2-(methylthio)ethyl)pyrrolidinium Chloride (**ESMmPyrrCl**)

1-Methylpyrrolidine (5.00 g, 0.0587 mol) was reacted with one equivalent of 2-chloroethyl methyl sulfide (7.32 g, 0.0587 mol) in a round-bottom flask with 30 mL of acetonitrile. The reactants were added under N_2_, and the flask was then sealed. The reaction was set up in an ice bath and stirred for 10 days at room temperature. The reaction mixture was then rotary evaporated to remove residual solvent, and the resulting pale-yellow crystals were isolated by vacuum filtration while washing with 200 mL of ethyl acetate and 40 mL of diethyl ether in a moisture-free tent. The resulting product was an ivory-colored crystalline solid (10.20 g, 89%, molar mass 195.01 g/mol). ^1^H (400 MHz; DMSO-*d*_6_) δ 2.09 (s, 4H), 2.15 (s, 3H), 2.87–2.92 (t, 2H), 3.01 (s, 3H), 3.48 (t, 4H), 3.53–3.57 (t, 2H); ^13^C (100 MHz; DMSO-*d*_6_) δ 14.60, 21.04, 26.07, 47.54, 61.93, 63.43.

#### Synthesis of 1-Methyl-3-(2-(methylthio)ethyl)imidazolium Chloride (**ESMmimCl**)

1-Methylimidazole (9.92 g, 0.121 mol) was reacted with 1.2 equivalents of 2-chloroethyl methyl sulfide (16.03 g, 0.145 mol) in a three-neck round-bottom flask with 60 mL of acetonitrile. The reaction was stirred for 30 min at 0 °C while purging with N_2_. It was then left to stir at 55 °C for 2 weeks. The reaction mixture was concentrated in vacuo. The resulting product was a pale-yellow liquid (23.67 g, >99%, molar mass 192.71 g/mol). ^1^H (400 MHz; DMSO-*d*_6_) δ 2.10 (s, 3H), 2.94–2.97 (t, 2H), 3.89 (s, 3H), 4.39–4.42 (t, 2H), 7.75 (d, 1H), 7.85 (d, 1H), 9.35 (s, 1H); ^13^C (100 MHz; DMSO-*d*_6_) δ 14.17, 32.92, 35.73, 47.35, 122.44, 123.49, 136.94.

### Synthesis of Thioether Ionic Liquids

#### Synthesis of 1-Methyl-1-(2-(methylthio)ethyl)pyrrolidinium Bis(trifluoromethylsulfonyl)imide (**ESMmPyrrNTf**_2_)

1-Methyl-1-(2-(methylthio)ethyl)pyrrolidinium chloride (12.32 g, 0.0632 mol) was reacted with 5% excess of lithium bis(trifluoromethylsulfonyl)imide (19.05 g, 0.0663 mol) dissolved in 30 mL of distilled water. The reaction mixture was left to stir at room temperature for 24 h. The product was washed with distilled water until the wash tested negative for chloride with 50 mM aqueous silver nitrate. The product was then rotary evaporated and placed in a high-vacuum oven at 55 °C for several days. The final product was a golden-colored liquid (10.69 g), 60%, molar mass 437.17 g/mol, water content: 82.6 ppm ^1^H (400 MHz; DMSO-*d*_6_) δ 2.08(s, 4H), 2.14 (s, 3H), 2.87–2.91 (t, 2H), 3.01 (s, 3H), 3.47 (t, 4H), 3.51-3.55 (t, 2H); ^13^C (100 MHz; DMSO-*d*_6_) δ 14.57, 21.04, 26.06, 47.51, 47.55, 61.95, 63.45, 117.86, 121.06.

#### Synthesis of 1-Methyl-3-(2-(methylthio)ethyl)imidazolium Bis(trifluoromethylsulfonyl)imide (**ESMmimNTf**_2_)

1-Methyl-3-(2-(methylthio)ethyl)imidazolium chloride (9.09 g, 0.0472 mol) was reacted with an equivalent of lithium bis(trifluoromethylsulfonyl)imide (13.5 g, 0.0472 mol) dissolved in 30 mL of distilled water. The reaction mixture was left to stir at room temperature for 24 h. The product was washed with distilled water until the wash tested negative for chloride with 50 mM aqueous silver nitrate. The product was then rotary evaporated and placed in a high-vacuum oven at 55 °C for 2 weeks. The final product was a colorless liquid (17.0 g), 83%, 437.42 g/mol, water content: 44.78 ppm, ^1^H (400 MHz; DMSO-*d*_6_) δ 2.09 (s, 3H), 2.93–2.96 (t, 2H), 3.88 (s, 3H), 4.36–4.40 (t, 2H), 7.70–7.71 (d, 1H), 7.77–7.78 (d, 1H), 9.14 (s, 1H); ^13^C (100 MHz; DMSO-*d*_6_) δ 14.10, 32.91, 35.71, 47.39, 114.64, 117.84, 121.04, 122.41, 123.52, 124.24, 136.80. Elemental analysis, calculated (%) for C_9_H_13_N_3_S_3_F_6_O_4_ (437.4 g/mol): C 24.75, H 3.27, N 9.52, F 25.87, S 22.00; Found: C 24.71, H 3.00, N 9.61, F 26.06, S 21.99.

### Physical Measurements

The water contents of the ionic liquids were measured with a Mettler Toledo DL39 coulometric Karl Fischer titrator. The water contents of the samples used for the property measurements ranged from 44 to 97 ppm.

### Viscosities

Viscosities were measured with a Cambridge Applied Systems ViscoLab 4100 electromagnetic reciprocating piston viscometer that was temperature-regulated by a Lauda RM-6 circulating bath with a 70/30 v/v propylene glycol/water mixture, as described in ref (27). Viscosities for each IL were recorded at intervals between 1 and 95 °C in ascending and descending order within their liquid ranges. The data were fit using the logarithmic form of the Vogel–Tammann–Fulcher relation
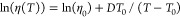
1in order to obtain better fitting of points in the low-viscosity range. Each data set included a value of 1 × 10^13^ cP at the liquid’s glass-transition onset temperature (*T*_g_), as described in ref (27). Values of ln(η_0_), *D*, and *T*_0_ are provided in the Results and Discussion section.

### Ionic Conductivities

Conductivity measurements were performed with a YSI model 3200 conductivity instrument fitted with a YSI model 3253 probe, housed within the same moisture-controlled dry box used for the viscosity measurements.

### Thermal Profiles

A PerkinElmer Pyris Diamond differential scanning calorimeter (DSC) with a liquid nitrogen cooling system was used to determine thermal profiles and glass-transition onset temperatures. Scan rates were 5 °C/min. The data were analyzed using Pyris software. The two thioether ionic liquids reported here were not observed to crystallize during triplicate DSC scans down to 153 K.

### Densities

Densities were measured gravimetrically using calibrated 1 mL volumetric flasks.

### Small-Angle X-ray Scattering (SAXS) Measurements

Approximately 0.2 mL of each IL sample was loaded into NMR tubes inside a glovebox (Ar Atmosphere). These NMR tubes are made with borosilicate glass, type 1 class A, and have an outer diameter of 2.99 ± 0.03 mm and were purchased from Sigma-Aldrich. Tubes were sealed with Teflon tape, removed from the glovebox, and immediately flame-sealed. SAXS experiments were carried out at Advanced Photon Source (APS) beamline 11-ID-C (Argonne National Laboratory, Lemont, IL), using an X-ray beam of energy 105 keV (λ = 0.1173 Å). The beam size of 0.5 mm × 0.5 mm was collimated by using a Laue monochromator. The diffraction images were collected using a PerkinElmer XRD1621 amorphous silicon 2D area detector. The sample detector distance for the experiments was set at a distance of 1600 mm, which covered a *q*-range from 0.2 to 6.5 Å^–1^, respectively, and was calibrated by using standard reference material (Si SRM640c). Here, q is the magnitude of momentum transfer X-ray scattering wave vector and is defined as , where 2θ represents the scattering angle and λ is the X-ray wavelength. The X-ray exposure time for each sample was 1 s per frame, and a total of 180 frames were collected. All of the measurements were performed at 300 K. We used the GSAS-II software^47^ to integrate the raw data to obtain intensity vs *q*.

The raw X-ray scattering intensity from each sample was corrected by subtracting the background intensity from an empty tube, as well as contributions from Compton, multiple, and diffuse scattering to obtain a coherent intensity it *I*_coh_(*q*) using *PDFgetX2*.^48^*S*(*q*) was calculated using the equation
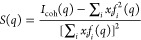
2where *i*, *x_i_*, and *f*_*i*_ represent the atomic species, atomic fraction, and X-ray form factor,^49^ respectively.^42,46,50^

### Computational Methods

All molecular dynamics simulations were carried out using the GROMACS software package version 4.5.5.^51^ For all our trajectories, the cutoff for all nonbonded interactions (Lennard Jones and Electrostatic) was set to 1.5 nm. The particle mesh Ewald^52,53^ method with a sixth-order interpolation and Fourier spacing of 0.08 nm was used for the long-range Electrostatic interactions. 3D periodic boundary conditions were used as coded in the GROMACS software. The force field parameters used in modeling the ionic liquids were based on OPLS-AA,^54^ Canongia Lopes and Pádua,^55−57^ Shimizu et al.,^58^ and Köddermann^59^ modifications of the Lennard Jones parameters for the anion and the imidazolium cation ring carbons and hydrogens. The bonds, angles, and torsion parameters involving oxygen and sulfur atoms were taken from OPLS-AA,^54^ Weiner,^60^ and Cornell et al.^61^ Alkane torsions were taken from Price et al.^62^ The atomic partial charges for the ether and thioether cations were fitted to the electrostatic potential surface in CHELPG using the DFT method and the B3LYP/6-311G(d,p) basis set in Gaussian-09;^63^ charges on equivalent atoms were averaged. Parameters are provided in sample GROMACS topology files in the Supporting Information.

Cubic configuration simulation boxes each containing 512 ion pairs were packed and energy-minimized. Our system size was chosen to balance the cost of computing dynamical properties and structural properties for systems where low-q features are not expected. Three equilibration steps were carried out in the NPT ensemble using the V-rescale thermostat^64^ and Berendsen barostat^65^ with 0.2 and 1.0 ps time constants, respectively. The first step, run at a pressure of 50 bar and with charges scaled to 1% of their target value was 0.2 ns in duration; the second period was 2 ns in duration with a pressure of 50 bar and charges at 10% of their target value, whereas the third period was 2 ns in duration, the pressure was 1 bar, and charges were set to 100% of their target value. This was followed by an 8 ns simulated annealing in the same ensemble from 300 to 600 K and back to the target temperature of 400 K. The final configuration of the annealing step was used for a 5 ns production run in the NPT ensemble using the Nose–Hoover thermostat^66^ and Parrinello–Rahman barostat^67^ with 0.2 and 1.0 ps time constants, respectively. Static structure functions *S*(*q*) were computed using the last 1 ns of the 5 ns production run.

To compute the cationic head–anion subcomponent of *S*(*q*, *t*) (vide infra), a 20 ns production run in the NVE ensemble was carried out, and to calculate the viscosity (η), for each system, 75 independent trajectories (7 ns each) were simulated in the NVT ensemble with the average temperature of the NVE run. These NVT simulations used the Nose–Hoover thermostat^66^ with a time constant of 0.5 ps and the velocity-verlet^68^ MD integrator. We used the protocol of Maginn et al.^69^ in which the Green–Kubo expression for the viscosity in eq 3 is used to generate an ensemble of functions ζ(*t*), where ⟨λ^*zx*^(0)λ^*zx*^(*t*′)⟩ corresponds to the stress tensor correlation function^70^
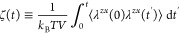
3These functions are used in two ways, first to obtain an average ⟨ζ(*t*)⟩ and second to obtain the time-dependent standard deviation of ζ(*t*), σ(*t*), across trajectories. A fit of σ(*t*) to the form σ_Fit_(*t*) = *Ã*·*t*^*b*^ is then carried out, as the Maginn algorithm^69^ to obtain the most accurate value of the shear viscosity requires finding first *t*_cut_, the time point at which the error σ_Fit_(*t*) is about 40% of an estimated viscosity value derived from the flat portion of ⟨ζ(*t*)⟩. The value of *t*_cut_ together with the function σ_Fit_(*t*) are then used as the upper bound and as the error for an error-weighted fit of ⟨ζ(*t*)⟩ using the expression in eq 4 from which the long time value is taken as the computed value of the shear viscosity η. In the Results and Discussion section where we refer to a normalized plot of the viscosity, what is meant is ⟨ζ(*t*)⟩_Fit_/η.
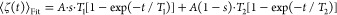
4All of the simulation work described in this article was carried out at a target temperature of 400 K except that to compare with experimental viscosity values at 325 K for the imidazolium-based ILs an extra set of 50 NVT trajectories equilibrated using the same protocols were run.

Equation 5 defines *S*(*q*, *t*)^1−3^
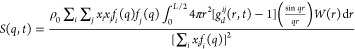
5Here, ρ_0_ is the total number density of the IL, *x_i_* and *x*_*j*_ are atomic mole fractions, and the *f*_*i*_(*q*) and *f*_*j*_(*q*) functions are the corresponding X-ray atomic form factors. In all our calculations, we use a Lorch-type function,^71,72^*W*(*r*), to account for the fact that box sizes are not infinite, resulting in reciprocal space oscillations due to cutoff errors in the normalized distinct van Hove function, *g*_d_^*ij*^(*r*, *t*), at the edge of the box. *g*_d_^*ij*^(*r*, *t*) was computed including periodic boundary conditions using an in-house modified version of the LiquidLib toolbox.^73^ The radial distribution function *g^ij^*(*r*) used for static structural calculations is simply *g*_d_^*ij*^(*r*, *t* = 0). *g*_d_^*ij*^(*r*, *t*) is equal to 1 at long times, and at any time it goes to 1 at long distances. Just as we have done in several prior publications, S(*q*) and *S*(*q*, *t*) can be partitioned into useful subcomponents.^1−3,5,38−41,43,45,46,74−85^ These allow us to follow the structural dynamics of liquid structural motifs at specific *q* values. In the Results and Discussion section, we will plot the time integral of the head–anion subcomponent of *S*(*q*, *t*) squared (*S*^H–A^(*q*, *t*)^2^). When describing these integrals as normalized, this will correspond to the function α^H–A^(*q*, *t*) defined in eq 6:
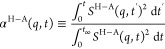
6A plot of α^H–A^(*q*, *t*) goes to 1 at long times, and its functional form can be directly compared with ⟨ζ(*t*)⟩_Fit_/η to establish connections between structural and viscoelastic relaxation times.^1,2^

## Results and Discussion

**Figure 3:**
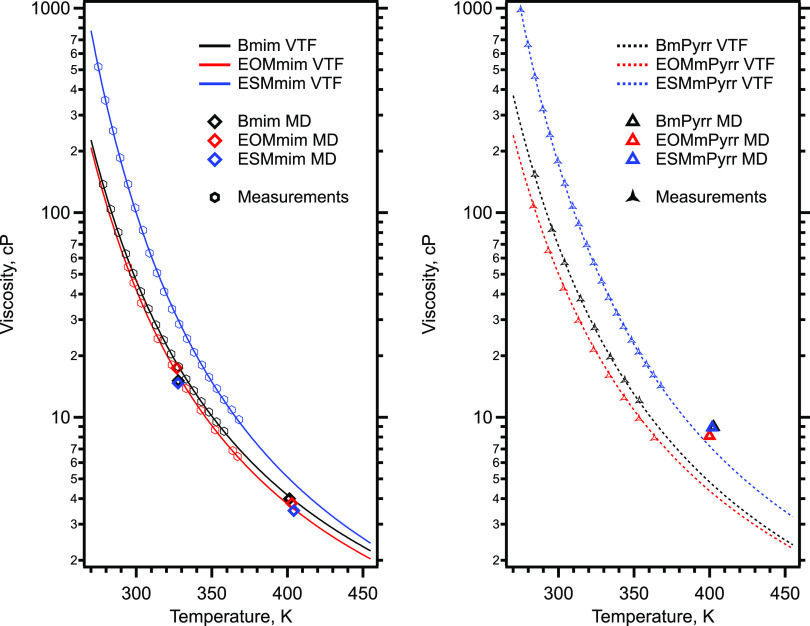
(Left) For the family of imidazolium-based ILs coupled with NTf_2_^–^, experimentally determined viscosity values, VTF fits of the experimental data that are extrapolated out to the temperature of the MD simulations, and the simulated viscosities. Measurements for Bmim are from Tariq et al.^24^ (Right) Same as left but for the pyrrolidinium-based ILs coupled with NTf_2_^–^.

The current study specifically focuses on NTf_2_^–^-based ILs coupled with the Bmim^+^, BmPyrr^+^, EOMmim^+^, ESMmim^+^, EOMmPyrr^+^, and ESMmPyrr^+^ cations (a schematic view of which is presented in Figure 1). Table 1 shows experimentally derived viscosity values at different temperatures as well as those obtained from our simulations; conductivities, *T*_g_, densities, as well as parameters to fit the viscosity dependence with temperature (see eq 1) are provided in Table 2. The data shows that for the six systems studied here, the pyrrolidinium-based ILs are more viscous than their imidazolium-based counterparts. Viscosity is one of the hardest quantities to converge in simulations of ILs (see the Methods section for a description of how costly these computations are in order to achieve accurate results) and for the most part, using the Maginn et al.^69^ methodology to predict these, the models used here appear to do a good job at capturing viscosity values. The key trend that we are seeking to study, which is the difference in viscosity across unsubstituted imidazolium and pyrrolidinium ILs or those with the exact same substitution, is always correctly captured by our simulations giving confidence in our analysis. To put this into perspective, Figure 3 shows experimentally measured values of the viscosity, fitted curves, and simulated results.

**Table 1:** Ionic Liquid Dynamic Viscosities Calculated from MD Simulation Trajectories Compared with Fits of Experimental Measurements Calculated at *T* = ⟨*T*_NVE_⟩^a^

	*T*_Sim._ (K)		η (cP)	
cation	*T*_target_	⟨*T*_NVE_⟩	η_Sim._	η_Expt-Fit_
Bmim^+^	325	327.7	15.1	17.8
	400	401.3	4.0	4.1
EOMmim^+^	325	326.7	17.4	16.6
	400	402.8	3.8	3.7
ESMmim^+^	325	327.6	14.7	30.0
	400	404.2	3.5	4.8
BmPyrr^+^	400	402.6	9.0	4.7
EOMmPyrr^+^	400	399.9	8.1	4.4
ESMmPyrr^+^	400	401.6	8.9	7.0

a Parameters for the VTF equation are given in Table 2.

**Table 2:** Physical Properties of the Ionic Liquids and the VTF Parameters Used for Fitting the Dynamic Viscosity

cation	*T*_g_ (K)	viscosity (cP, 25 °C)	conductivity (mS/cm, 25 °C)	density (g/cm^3^, 22 °C)	ln(η_0_) (cP)	*D*	*T*_0_ (K)
Bmim^+^	186	50	3.3	1.44	–1.93	4.99	160.8
EOMmim^+^	188	45	4.6	1.45	–1.95	4.74	163.7
ESMmim^+^^a^	198	110	2.0^b^	1.50	–2.21	5.17	170.6
BmPyrr^+^	184	75	2.7	1.39^c^	–2.28	6.11	154.1
EOMmPyrr^+^	184	54	3.9	1.46^d^	–2.06	5.59	155.1
ESMmPyrr^+^^a^	200	191	1.2^b^	1.45^e^	–2.21	5.73	168.7

a This work. For all other data, see ref (27).

b Measured at 24 °C.

c Measured at 23 °C.

d Measured at 20 °C.

e Measured at 25 °C.

Perhaps a surprising finding is that while still following this trend, the S-substituted ILs are significantly more viscous than the O-substituted and the unsubstituted (alkyl) analogues. This is a trend that the simulations do not appear to capture. It is likely that the issue stems from the fact that the S atom in ESMmim^+^ and ESMmPyrr^+^ is polarizable and fixed-charge models simply do not capture its fluctuating charge. Empirically speaking, the higher viscosities of the thioether congeners that are observed in the range of normal working temperatures are related to the fact that their glass-transition temperatures are 10–16 K higher than the alkyl and ether derivatives for a given cation family. Within each family, the fragilities (deviations from Arrhenius behavior as quantified by the *D* parameter in VTF fitting) and limiting viscosities at infinite temperature (η_0_) only vary over small ranges, but the differences in *T*_g_ (and consequently *T*_0_) significantly influence the respective viscosity behaviors with temperature. The thioether groups apparently have a very large impact on the conformational dynamics of the cations at low temperatures, which would be very interesting but difficult to simulate because of the ergodicity problems associated with the high viscosity.

**Figure 4:**
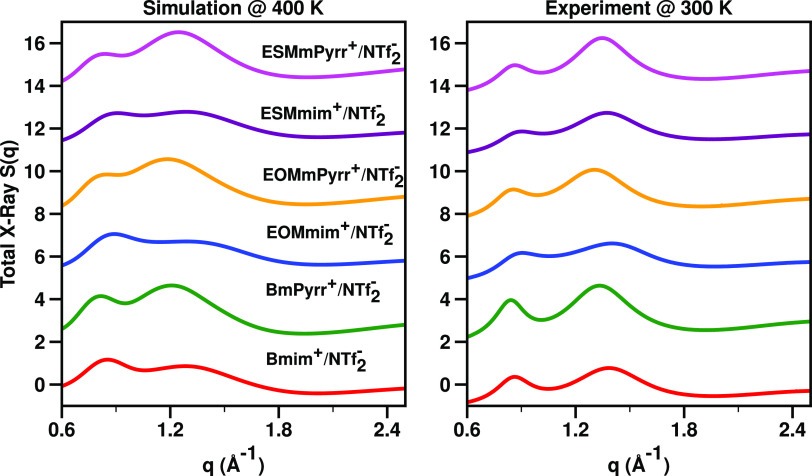
(Right) Experimental small-angle X-ray scattering total structure function *S*(*q*) at 300 K. (Left) Simulated *S*(*q*) at 400 K. Both figures show in the region above 1 Å^–1^ and below 1.8 Å^–1^ what we call an adjacency peak; they also show at around 0.8 Å^–1^ the charge alternation peak associated with the charge trio discussed in Figure 5.

From a static structural perspective, the ILs studied here share many similarities, as can be gleaned from a comparison of the SAXS and simulated structure functions *S*(*q*) in Figure 4. None of these systems have large tails that would cause significant charge-tail segregation, and a pre-peak is not expected. For all of the ILs studied here, the peak at about 0.8 Å^–1^ is due to the 
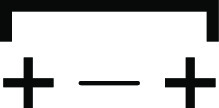
 and 
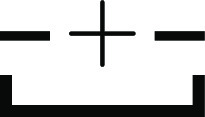
 motif along charge networks, i.e., it corresponds to the typical separation between the cationic heads (or the anions) spaced by ions of opposite charge (the anions or the cationic heads, respectively). In contrast, the peak above 1 Å^–1^ is due to all sorts of inter- and intramolecular adjacency correlations.^5,38−41,43,45,46,74−79^

**Figure 5:**
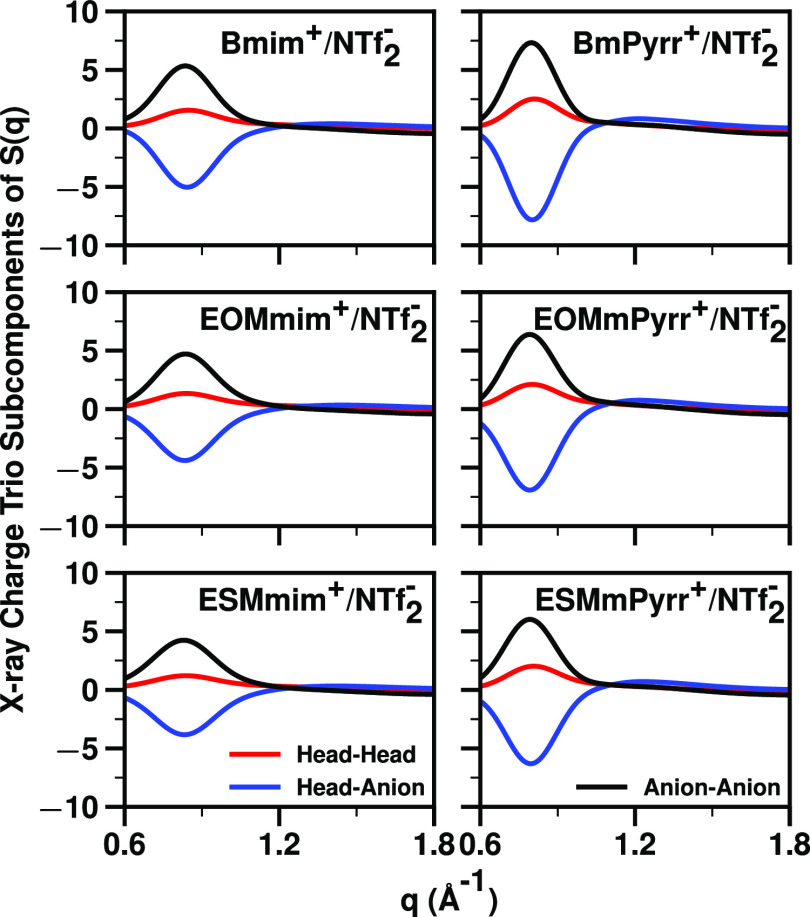
For all ILs, charge trio subcomponents of *S*(*q*) in the relevant charge alternation q-regime at 400 K.

Figure 5 shows sets of computationally derived subcomponents of *S*(*q*), specifically the “charge trio” head–head (*S*^H–H^(*q*)), anion–anion (*S*^A–A^(*q*)), and head–anion (*S*^H–A^(*q*)) with species defined in Figure 1; for a description of the many ways one can partition the total *S*(*q*) of ILs into subcomponents, see refs^5,38−41,43,45,46,74−79^ and citations therein. The charge trio subcomponents of *S*(*q*) are particularly important because at about 0.8 Å^–1^ they carry information about the structure of the charge network (
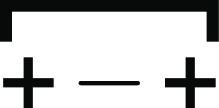
 and 
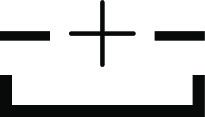
 correlations).^5,38−41,43,45,46,74−86^ In the region 1–1.8 Å^–1^, only one of these three subcomponents, namely, *S*^H–A^(*q*), is most relevant as it provides direct information about the very important adjacency correlations associated with positive and negative 
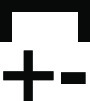
 interactions along the charge network. *S*^H–H^(*q*) and *S*^A–A^(*q*) are less important in the q-regime associated with adjacency correlations because, except for intramolecular interactions, species with identical charge are not expected to be adjacent. The first thing to notice in Figure 5 is that in the charge alternation regime around 0.8 Å^–1^ the intensities of the subcomponents, particularly those involving the high X-ray contrast anion (*S*^A–A^(*q*) and *S*^H–A^(*q*)) are very large compared to that of the overall *S*(*q*) in the same *q*-regime. In this regime, *S*^A–A^(*q*) and *S*^H–H^(*q*) always show as peaks, whereas *S*^H–A^(*q*) always shows as an antipeak; for all ionic liquids and molten salts, this behavior is the hallmark of charge alternation.^5,38−41,43,45,46,74−85^ In this q-regime, the overall *S*(*q*) signal is to a large extent the result of massive cancellations due to the interference of *S*^A–A^(*q*), *S*^H–H^(*q*), and *S*^H–A^(*q*). Because of these cancellations of large numbers, it is not uncommon to find in this *q*-region that small flaws in force fields give rise to inaccuracies in the overall *S*(*q*) compared to experiments.

Most interesting in Figure 5 is that when we compare each imidazolium-based IL with the analogous pyrrolidinium-based system, the peaks and antipeaks associated with this trio at around 0.8 Å^–1^ are more pronounced for pyrrolidinium across the whole series (for each IL, compare the left and right panels in Figure 5). Since (i) the anion in all cases is the same, (ii) H atoms have small X-ray contrast, and (iii) the contrast difference between a single C atom and a N atom is not large, we cannot but conclude that such differences in intensity must be due to bonafide liquid structural differences and not due to trivial issues of contrast. In other words, the significant differences going from left to right in each panel of Figure 5 must arise because the pyrrolidinium-based cations have a better-defined/structured charge network.

**Figure 6:**
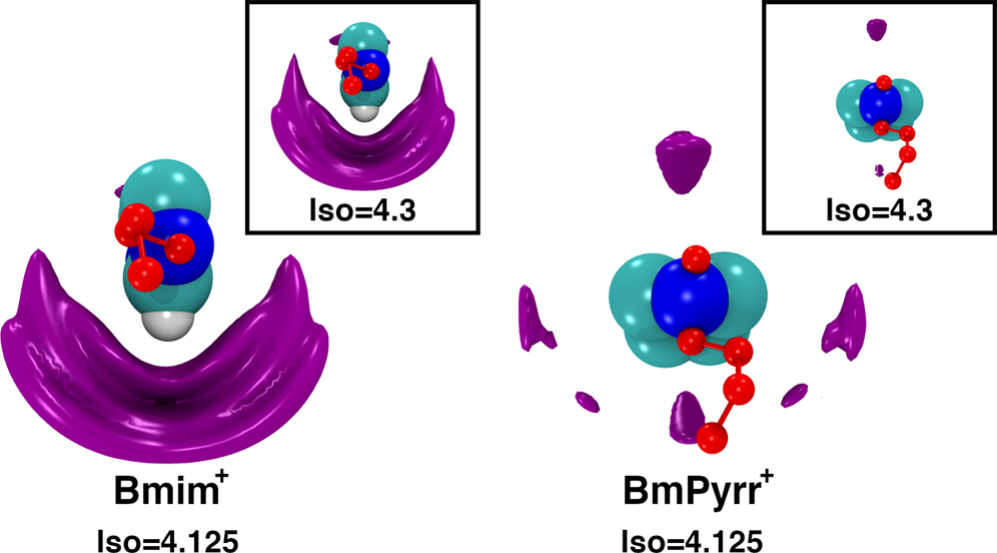
Spatial distribution functions computed with the TRAVIS software^87^ for atoms in NTf_2_^–^ around Bmim^+^ and BmPyrr^+^ at 400 K. Left and right panels are at the same isovalue. We see from these figures that there is a large and directional probability of finding anions on one side of the Bmim^+^ ring, but the distribution around BmPyrr^+^ is much more symmetrical and multidirectional. When the isovalue is increased in the inset, the figure for Bmim^+^ almost does not change, whereas BmPyrr^+^ has almost no contributions at this level highlighting the difference in the angular accumulation of the anions around each of the cations.

For the case of Bmim^+^/NTf_2_^–^ and BmPyrr^+^/NTf_2_^–^, in Figure 6 we show spatial distribution functions of atoms in the anion around the cationic ring. Figure 6 shows that for a fixed large value of the anionic isodensity (in units of atoms per unit volume), the anions are very asymmetrically distributed around the imidazolium ring, covering only the side where the most acidic proton is located, between the two nitrogen atoms. If we choose an even larger value of the isodensity, we still see significant space covering for the imidazolium-based IL, but almost no covering in the case of the pyrrolidinium-based IL. In other words, anions are spatially spread in the case of BmPyrr^+^/NTf_2_^–^ with the anionic density distribution around the pyrrolidinium ring looking somewhat tetrahedral; no particular spot around the cation carries a distinctly large isodensity associated with highly directional accumulation of anions, and the opposite is true for the case of Bmim^+^/NTf_2_^–^. We do not know if this specific topological difference is associated with differences in viscosity or the viscoelastic relaxation, but it certainly is suggestive of very different orientational requirements in each case. Seen as a whole, Figure 6 shows that for BmPyrr^+^/NTf_2_^–^, anions can anchor the cation in multiple directions, whereas in the case of Bmim^+^/NTf_2_^–^, the charge network is highly constrained to accommodate directional ordering.

We now discuss the time dependence of the charge alternation and adjacency structural motifs and attempt to link these to the viscoelastic relaxation. In previous studies,^1−3^ we have shown that the simplest and best way to do this is by following the structural dynamics of the cationic head–anion subcomponent *S*^H–A^(*q*, *t*). Because simulations do not accurately capture the viscosity trend for the S-substituted ILs along a family (the trend between families is well captured), we focus our attention on the ILs with the Bmim^+^, BmPyrr^+^, EOMmim^+^, and EOMmPyrr^+^ cations. Through prior work, we have learned^1−3^ that the relaxation time of *S*^H–A^(*q*, *t*) in the different relevant q-regimes associated with adjacency and charge alternation closely matches that of the overall *S*(*q*, *t*) but *S*^H–A^(*q*, *t*) has the unique advantage that its interpretation in terms of the IL structural motifs is clear and not polluted by a myriad of other contributions that are difficult to disentangle. This function captures in its most accurate form the dynamics of charge alternation as well as the specific adjacency correlations associated with cationic heads and anions. If an IL were to also have a first sharp diffraction peak due to tail-charge network alternations, at low *q*, *S*^H–A^(*q*, *t*) would capture the 
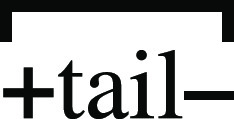
 and 
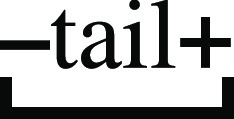
 dynamics that are representative of across-charge-network processes, as opposed to along-charge-network dynamics. Such across-network processes include rare-event network swaps and other slow events.

**Figure 7:**
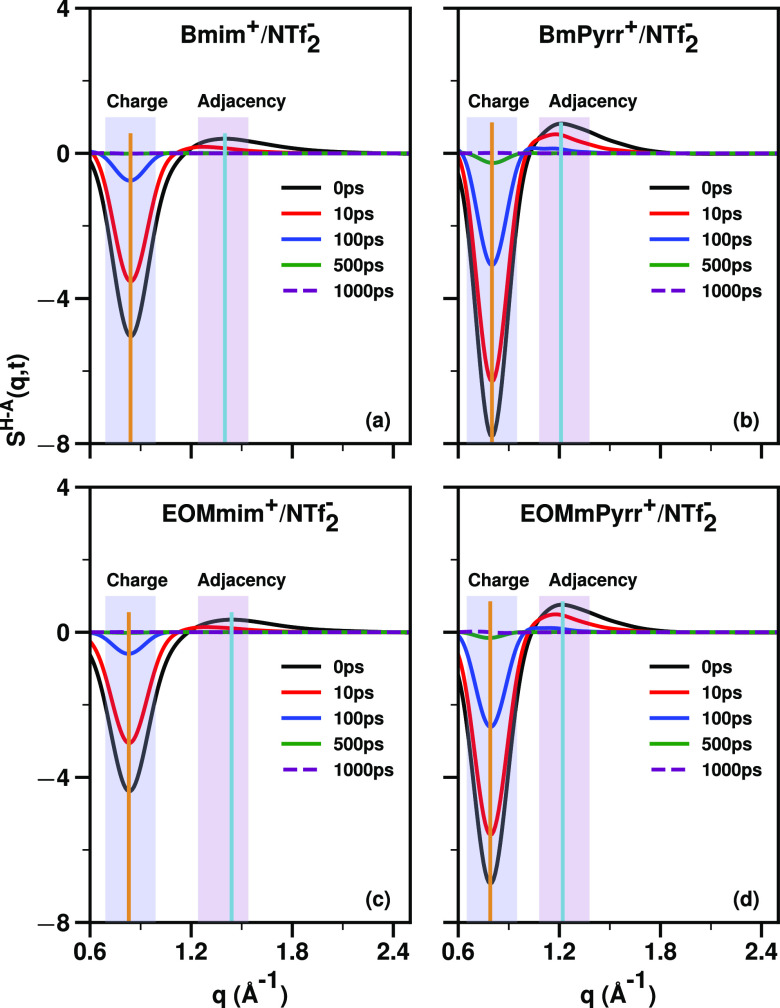
For NTf_2_^–^ coupled with Bmim^+^, BmPyrr^+^, EOMmim^+^, and EOMmPyrr^+^, the *S*^H–A^(*q*, *t*) subcomponent of *S*(*q*, *t*) at 400 K.

**Figure 8:**
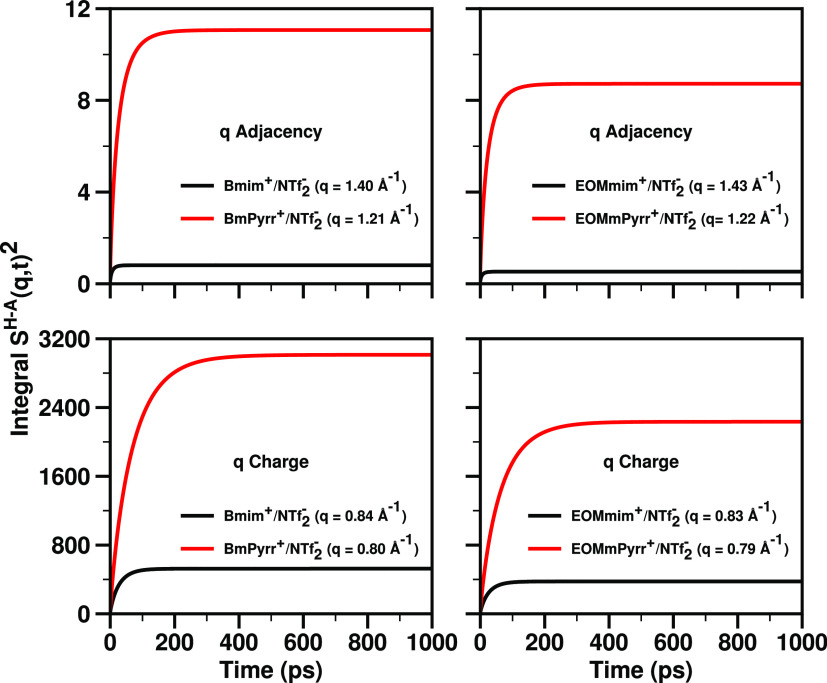
For ILs of Bmim^+^, BmPyrr^+^, EOMmim^+^, and EOMmPyrr^+^ coupled with NTf_2_^–^, the running integral at 400 K of *S*^H–A^(*q*, *t*)^2^ (∫_0_^*t*^*S*^H–A^(*q*, *t*′)^2^ d*t*′) at *q* values corresponding to the adjacency and the charge alternation structural motif as defined in Figure 7.

Figure 7 unequivocally shows that in the most relevant q-regime for viscosity (the “within-charge-network” structural relaxation region where *q* is ∼0.8 Å^–1^), *S*^H–A^(*q*, *t*) relaxes significantly faster for the imidazolium-based IL compared to the pyrrolidinium-based IL, and we see this also from the time at which the running integrals of the square of *S*^H–A^(*q*, *t*) become flat in Figure 8. These findings appear to provide a quantifiable physical perspective on what hard and soft ions do differently on a structural basis. In this case, the pyrrolidinium-based cations are harder, resulting in: (1) ILs with a larger magnitude of the charge alternation trio, as can be gleaned from Figure 5, (2) slower in-network structural relaxation as seen from the time it takes to flatten the curves in Figure 7 and to converge those in Figure 8, as well as (3) having higher dynamic viscosity.

**Figure 9:**
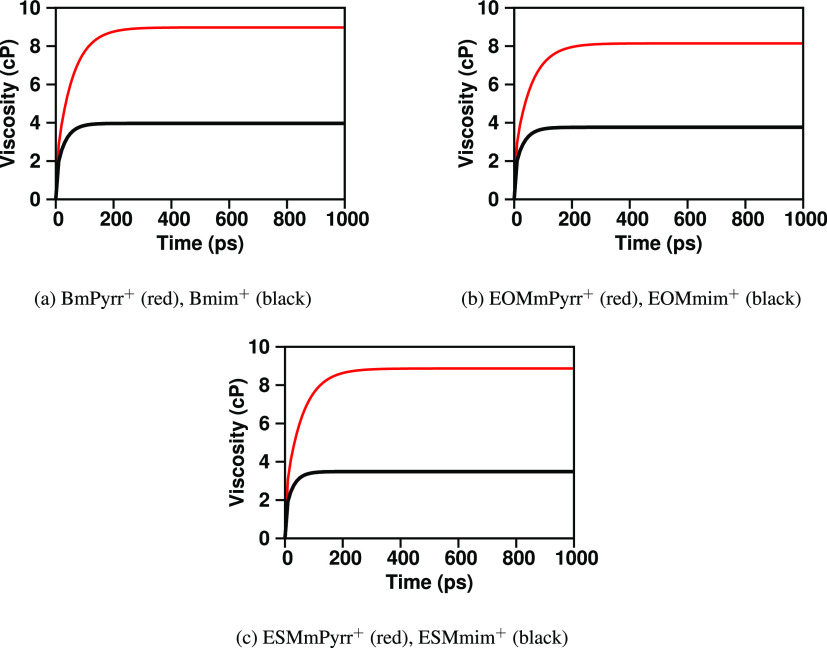
For NTf_2_^–^-based ILs coupled with different cations of the imidazolium and pyrrolidinium families, running integrals of the Green–Kubo expression, ⟨ζ(*t*)⟩_Fit_, for the viscosity using the Maginn et al. method^69^ of calculation at 400 K. The most important finding, besides the actual viscosity value derived at a long time in each case, is the time it takes for each of these functions to converge to a flat value. In all cases, not only is the viscosity higher for the pyrrolidinium-based ILs but also the time it takes them to approach asymptotic values is always longer than for the imidazolium ILs.

**Figure 10:**
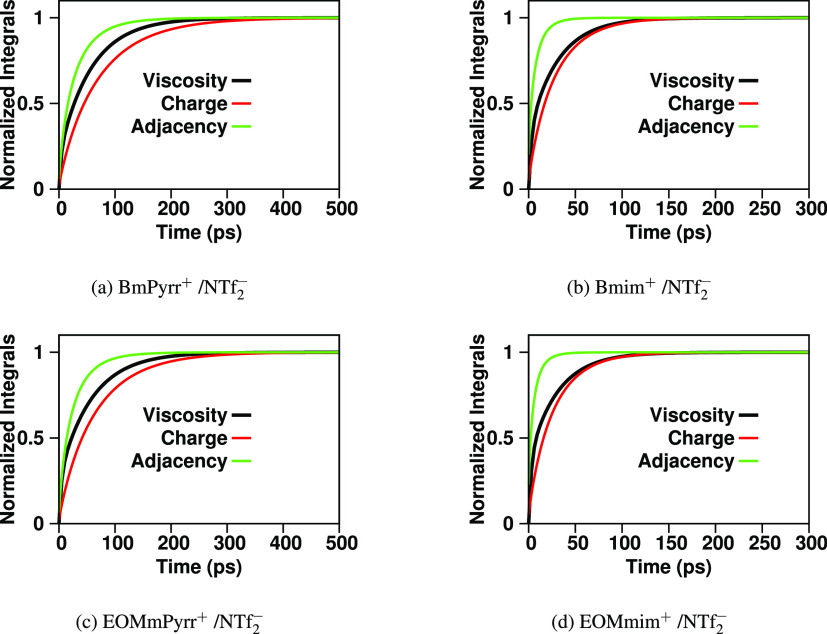
α^H–A^(*q*, *t*) (the normalized integral of *S*^H–A^(*q*, *t*)^2^ as defined in the Methods section), compared to the normalized viscoelastic relaxation ⟨ζ(*t)*⟩_Fit_/η at 400 K. In all cases, the relaxation of the viscosity falls in between that of adjacency and charge alternation correlations indicating that its origin is a combination of processes associated with both.

To also see this from the point of view of the viscoelastic relaxation, Figure 9 shows ⟨ζ(*t*)⟩_Fit_, our best estimate of the running integral corresponding to the Green–Kubo expression for the viscosity (see the Methods section for a detailed description of the error-weighted fitting scheme by Maginn and co-workers^69^). In all cases, not only is the actual viscosity larger for the pyrrolidinium family of ILs compared to the analogous imidazolium family member (the asymptotic value at a long time in Figure 9) but also the time to reach this asymptotic value is longer. This is exactly the same behavior we observe for the integrals of S^H-A^(*q*, *t*)^2^. Notice that slower viscoelastic relaxation does not always necessarily mean larger viscosity, but in this case, it does. To put this last finding into context, Figure 10 plots the normalized running integral of the viscosity, ⟨ζ(*t)*⟩_Fit_/η, together with those of *S*^H–A^(*q*, *t*)^2^ (the function α^H–A^(*q*, *t*) defined in the Methods section). As expected, the viscoelastic relaxation always falls somewhere between that of the fastest and slowest relevant structural IL motif; in our case, these are the relaxation of adjacency correlations and that of charge alternation. The most important point in this plot is how significantly longer each relaxation is in the case of the pyrrolidinium-based ILs compared to the same quantity or process for the imidazolium-based ILs. Each of our findings associated (1) with the viscoelastic relaxation, (2) the actual viscosity, (3) the dynamics of *S*^H–A^(*q*, *t*)^2^, and (4) the static magnitude of the trio components of *S*^H–A^(*q*) in the charge alternation regime is an alternative way to present a rigorous atomistic view of the hard/soft ionic concept.

## Conclusions

This article discusses a set of NTf_2_^–^-based ILs where the cations are Bmim^+^, EOMmim^+^, ESMmim^+^, BmPyrr^+^, EOMmPyrr^+^, and ESMmPyrr^+^ and provides novel physical data for the S-substituted ILs. An important finding from this work is that for ILs with similar X-ray contrast, we are able to make a link between cationic hardness and the intensity of certain subcomponents of *S*(*q*) in the charge alternation regime, the difference in structural relaxation times, the difference in viscoelastic relaxation times and also in their actual dynamic viscosities. We conclude that the combination of patterns for the pyrrolidinium-based ILs physically identifies them as the harder cations that necessarily create stiffer charge networks. Interestingly, the S-substituted ILs are more viscous than their counterparts, and there is evidence that trends we have described can be reversed if a single methylene bridge instead of an ethyl bridge is included separating the ring and the substituent. We plan to explore these intriguing differences in the pattern of viscosity in a future publication.

## Supporting Information Available

Bmim.itp, Bmim-NTf2.top, BmPyrr.itp, BmPyrr-NTf2.top, EOMmim.itp, EOMmim-NTf2.top, EOMmPyrr.itp, EOMmPyrr-NTf2.top, ESMmim.itp, ESMmim-NTf2.top, ESMmPyrr.itp, ESMmPyrr-NTf2.top, and NTf2.itp with parameters for ILs to be used with the software GROMACS (ZIP)
